# Low-dose interleukin 2 therapy halts the progression of post-streptococcal autoimmune complications in a rat model of rheumatic heart disease

**DOI:** 10.1128/mbio.03823-24

**Published:** 2025-02-25

**Authors:** Rukshan Ahamed Mohamed Rafeek, Natkunam Ketheesan, Michael F. Good, Manisha Pandey, Ailin Lepletier

**Affiliations:** 1 School of Science and Technology, University of New England, New South Wales, Australia; 2 Institute for Biomedicine and Glycomics, Griffith University, Gold Coast, Queensland, Australia; Max Planck Institute for Infection Biology, Berlin, Germany

**Keywords:** acute rheumatic fever, rheumatic heart disease, group A streptococcus, low-dose interleukin-2, immunotherapy, autoimmune diseases

## Abstract

Acute rheumatic fever (ARF) is an autoimmune disease triggered by antibodies and T cells targeting the group A *Streptococcus* (GAS, Strep A) bacterium, often leading to rheumatic heart disease (RHD) and Sydenham’s chorea. Long-term monthly penicillin injections are recognized as a cornerstone of public health programs to prevent Strep A reinfection and progression of ARF. However, compliance is poor, and better tools are required to slow disease progression. Preclinical evidence suggests that this can be achieved. Using a rat model that replicates post-streptococcal autoimmune complications, we explored the potential of low-dose interleukin-2 (LD-IL-2) as an immunotherapeutic intervention for ARF/RHD. In this model, injections of recombinant M protein from Strep A type 5 (rM5) to Lewis rats induce cardiac tissue inflammation, conduction abnormalities, and cross-reactive antibodies against cardiac and brain proteins central to disease pathogenesis. In animals injected with rM5 and treated with LD-IL-2, no cardiac functional or histological changes was observed. LD-IL-2 therapy effectively reduced the production of cross-reactive antibodies raised against host proteins and significantly increased regulatory T cells in the mediastinal lymph nodes. These novel findings suggest that LD-IL-2 will be an effective immunotherapeutic agent for treating ARF and has the potential to replace the standard monthly penicillin injections.

**IMPORTANCE:**

Post-streptococcal autoimmune syndromes, including acute rheumatic fever, rheumatic heart disease, and Sydenham’s chorea, represent a significant yet often under-recognized health and economic burden. This is especially true in low-income countries and among Indigenous populations in high-income nations, where the disease burden is most severe. These conditions arise from an autoimmune response to group A *Streptococcus* infections, leading to long-term health complications, disability, and premature death. Despite their widespread impact, no vaccine is currently available to prevent reinfections, and no specific therapy exists to treat the resulting autoimmune process. This study uses a rat model of rheumatic heart disease to evaluate the potential of low-dose interleukin 2 therapy in improving clinical outcomes and reducing the incidence of autoimmune diseases triggered by streptococcal infections.

## INTRODUCTION

Rheumatic heart disease (RHD) is a neglected disease of poverty that affects 40 million individuals worldwide and leads to more than 350,000 deaths annually. People in low- and middle-income countries and First Nations peoples in developed countries are principally affected (1). Indigenous Australians have some of the highest recorded rates of RHD (2). RHD accounts for nearly 2% of all deaths from cardiovascular diseases and is the commonest cause of pediatric-acquired heart disease (1).

In susceptible individuals, infections with group A *Streptococcus* (GAS, Strep A) initiate an autoimmune process that leads to acute rheumatic fever (ARF). Approximately 60% of patients who experience at least one episode of ARF will develop irreversible damage to the heart valves, which defines RHD (2). Besides, up to 30% of patients with ARF develop Sydenham’s chorea, a neurobehavioral condition characterized by involuntary choreiform movements and neuropsychiatric impairment that usually occurs in association with cardiac symptoms. Of the ARF diagnoses between 2016 and 2020 in Australia, almost half were children aged 5–14 years. ARF rates were generally higher in males under 15 years, while in adults, the rates were higher in females (3). Although ARF is a systemic disease affecting multiple organs, only carditis results in lifelong consequences.

RHD develops as a complication of ARF, where antibodies and T cells that target streptococcal proteins, including the M protein, also react with host heart proteins resulting in valvular damage. This process, known as molecular mimicry, is part of the host’s immune response to Strep A (4). The structural similarity between streptococcal M proteins and human proteins such as cardiac myosin, tropomyosin, and laminin is a key determinant for the development of RHD. Anti-streptococcal antibodies may initially target laminin in the valvular endothelium, exposing hidden epitopes on intracellular proteins and promoting the migration of inflammatory cells into the valves. This process results in the recognition of autoantigens, amplifying the inflammatory response and leading to tissue destruction and damage to the heart valves (5). Studies have identified T helper cells producers of IFN-ɣ and IL-17 (Th1 and Th17, respectively) as key mediators of RHD (6, 7). Additionally, patients with RHD exhibit a deficiency of regulatory T cells (Tregs), a specialized T cell subset that regulates the immune system maintaining homeostasis and self-tolerance (8, 9), along with an elevated Th17/Treg ratio. This imbalance is more pronounced in patients with multivalvular involvement compared to those with univalvular disease (10, 11).

Currently, there is no specific treatment to halt the progression of ARF to RHD. Additionally, there is no vaccine available to prevent repetitive Strep A infections, which can exacerbate ARF and drive the pathological process. For decades, patients diagnosed with ARF have been prescribed regular (four weekly) and long-term (up to 10 years or more) administration of penicillin to prevent recurrent Strep A infections (5, 12); however, poor compliance with this regimen highlights the need for better tools to manage Strep A autoimmune complications. Low-dose interleukin-2 (LD-IL-2) therapy is known for its safety and has shown efficacy in treating autoimmune conditions in some patients with type 1 diabetes (13), systemic lupus erythematosus (14), primary Sjögren syndrome (15, 16), rheumatoid arthritis (17), and vasculitis associated with chronic hepatitis C virus (18).

Unlike conventional immunosuppressive treatments, LD-IL-2 therapy promotes immune tolerance by specifically targeting Tregs while preserving effector immune cell function (19, 20). Five-day course of LD-IL-2 therapy with daily injections can harness IL-2’s immunomodulatory properties and mitigate autoimmune responses in humans and in preclinical models (21, 22). LD-IL-2 therapy has shown promise in several human trials and is increasingly seen as a viable treatment option for cases where conventional therapies fail. It has demonstrated positive results in lupus erythematosus, where it promoted proliferation and recovery of multiple subtypes of Tregs, including skin-homing Tregs interacting with endothelial cells (23, 24). LD-IL-2 treatment of patients with primary Sjögren syndrome resulted in a significant clinical improvement, restoring immune balance and metabolic pathways linked to changes in immune cell subsets, particularly the expansion of Tregs (15, 16). In rheumatoid arthritis, combining LD-IL-2 with tocilizumab was found to be safe and effective, reducing effector T cell levels while boosting Tregs, ultimately helping to prevent disease progression (17). Additionally, no severe adverse events have been reported in any of the patients treated with LD-IL-2 therapy to date. Nevertheless, variability in patient responses remains a challenge, likely linked to the heterogeneous nature of these diseases and to difficulties in defining the optimal “low-dose” range of IL-2 needed to effectively correct the imbalance between immune tolerance and autoimmunity.

In this study, we employ the rat autoimmune valvulitis (RAV) model to investigate whether LD-IL-2 therapy can be extended to treat active ARF and prevent its complications. The RAV model utilizes Lewis rats to identify key mechanisms in post-streptococcal autoimmune heart disease, including the cross-reactivity of antibodies with both bacterial and cardiac tissues, the role of T cells —particularly the Th17 phenotype— in cardiac inflammation, and the impact of anti-streptococcal antibodies on heart dysfunction (25–28). The histological, immunological, and functional changes in the hearts resemble those of RHD, including the presence of Aschoff’s nodules.

## RESULTS

**Fig 1:**
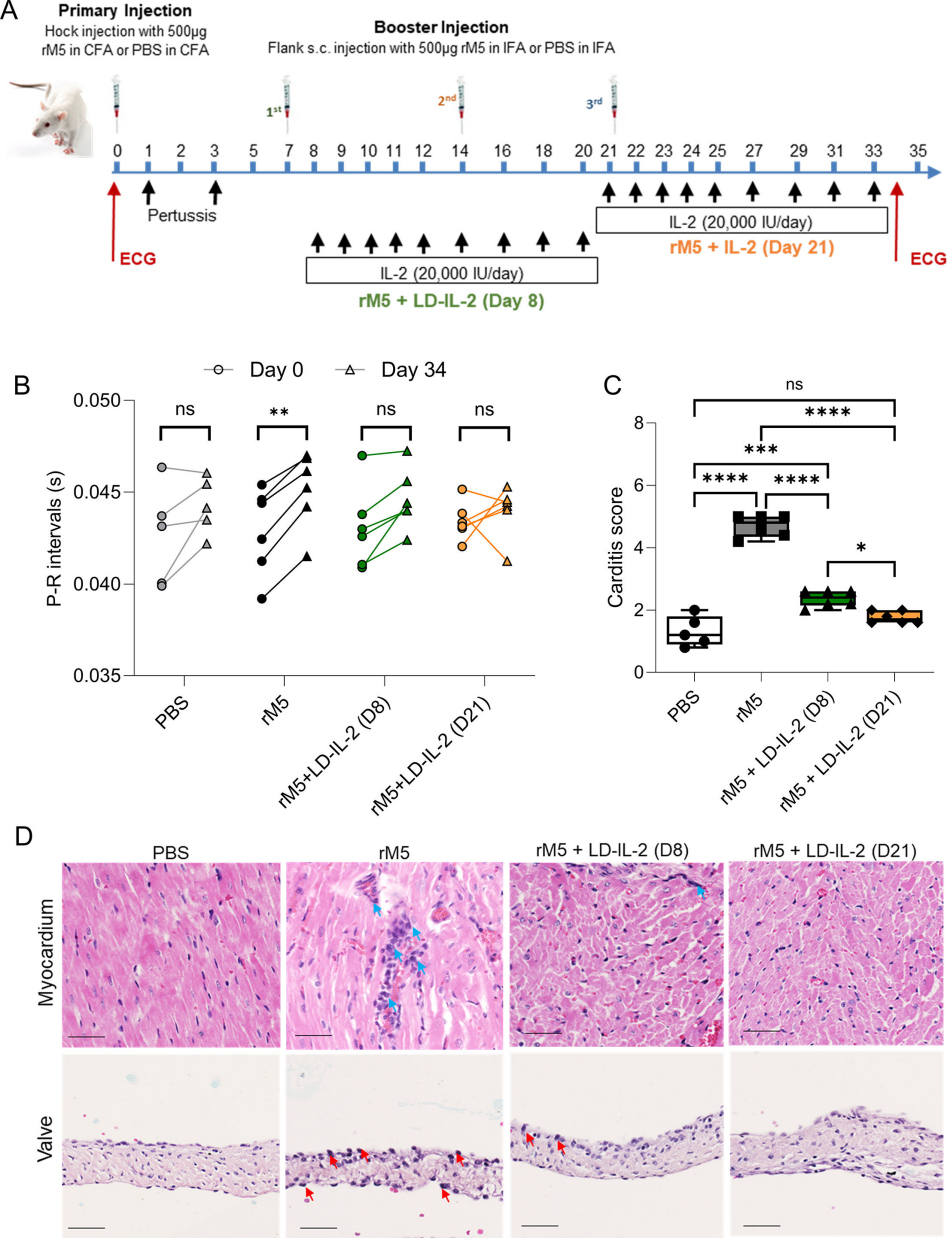
LD-IL-2 therapy induces functional improvement of the heart and reduces carditis. (A) Experimental timeline summarizing development of low-dose IL-2 (LD-IL 2) therapeutics for ARF/RHD using the rat autoimmune valvulitis model. Rats received 3 boosters with Strep A rM5 at weekly intervals. On Days 1 and 3, the rats were given 0.3 µg of *Bordetella pertussis* toxin via intraperitoneal injection. Subsequently, on Days 7, 14, and 21 following the initial injection, the rats received booster injections of either rM5 or phosphate-buffered saline (PBS) mixed with incomplete Freund’s adjuvant (IFA) subcutaneously in the flank. LD-IL-2 treatment began on either Day 8 (D8) or Day 21 (D21) post-primary injection with Strep A rM5. Rats were euthanized at Day 35 for assessment of carditis and immune responses. (B) Functional assessment of the heart before (•, Day 0) and after (∆, Day 34) injection of PBS or rM5 by ECG. Antigen-injected rats (n=5 6 females/grp) received LD-IL-2 therapy or were left untreated. Statistical analysis performed by two-way ANOVA. n.s. non-significant, ** p<0.01. (C) Inflammatory changes in the myocardium and valvular tissue, characterized by mononuclear cell infiltration, were scored in rats injected with PBS or rM5, which were either treated with LD-IL-2 or left untreated. Box-and-whisker plot shows min to max carditis scores within each treatment group. Statistical analysis for carditis score was performed by one-way ANOVA. n.s. non-significant, *P < 0.05, ***P < 0.005, ****P < 0.001. (D) Representative histological images of myocardium and valvular tissues. Arrows indicate inflammatory cell infiltration in the myocardium (blue arrows) and valves (red arrows). Scale bar = 50 µm.

To replicate the early events leading to the clinical syndromes of post-streptococcal autoimmune complications, we administered multiple subcutaneous injections of a recombinant M protein from Strep A type 5 (rM5) —Strep A M5 is a classical “rheumatogenic” strain— emulsified in Freund’s adjuvant to Lewis rats. In addition, on Days 1 and 3 after the primary injection, rats received intraperitoneal administration of *Bordetella pertussis* toxin, followed by booster injections of rM5 or phosphate-buffered saline (PBS) in incomplete Freund’s adjuvant on Days 7, 14, and 21, as shown in Fig. 1A. To investigate whether LD-IL-2 therapy can be repurposed to treat carditis, we compared rats injected with adjuvant and rM5 with control rats injected with just adjuvant and PBS, which were either treated with LD-IL-2 or left untreated. Treatment regimen consisted of repeated subcutaneous injections of LD-IL-2 after rats have received the first (Day 8) or last (Day 21) booster with rM5 (Fig. 1A). For both regimens, LD-IL-2 was administered daily for 5 days, followed by four injections with a 2-day interval between each injection. Euthanasia was performed on Day 35, either 15 days following the conclusion of LD-IL-2 treatment (Day 8) or 2 days after the end of treatment (Day 21).

### LD-IL-2 therapy prevents the functional and histological cardiac changes associated with RHD

An important characteristic of the RAV model is its ability to induce observable functional and histological changes to the heart, mirroring those clinically observed in patients with RHD. To measure conduction abnormalities and assess cardiac function, all rats underwent electrocardiography (ECG) before being euthanized. Baseline heart function prior to the first rM5 injection was used to compare functional changes. Peak values of *P* and *R* points at three different segments of ECG from each rat were individually measured and analyzed. A prolongation of the *P*–*R* interval was found in all rM5-injected rats, indicating a conduction abnormality in the heart (Fig. 1B). In contrast, no significant changes to the *P*–*R* interval were observed following either LD-IL-2 therapeutic regimen.

To investigate the effect of LD-IL-2 therapy on inflammatory changes associated with ARF/RHD, infiltrating mononuclear cells were assessed following hematoxylin and eosin (H&E) staining. The extent of inflammation in the myocardium and valves was expressed as a “carditis score”, which was based on the number of mononuclear cell infiltrates and focal lesions in both the myocardium and mitral valve. Carditis scoring was undertaken using a scoring matrix previously developed (26). The scoring system assessed valvular and myocardial sections from the different experimental groups. An increased mononuclear cell infiltration was observed in rats injected with rM5 compared to PBS control group. In contrast, minimal mononuclear cell infiltration was observed in rM5-injected rats treated with LD-IL-2 from either Day 8 or Day 21. Both LD-IL-2 treatment regimens led to significant reduction in carditis (Fig. 1C and D). Although the carditis scores were statistically different between rats treated using the Days 8 and 21 regimens, the absolute differences were minimal.

### Reduction of cross-reactive M protein antibodies following LD-IL-2 therapy

**Fig 2:**
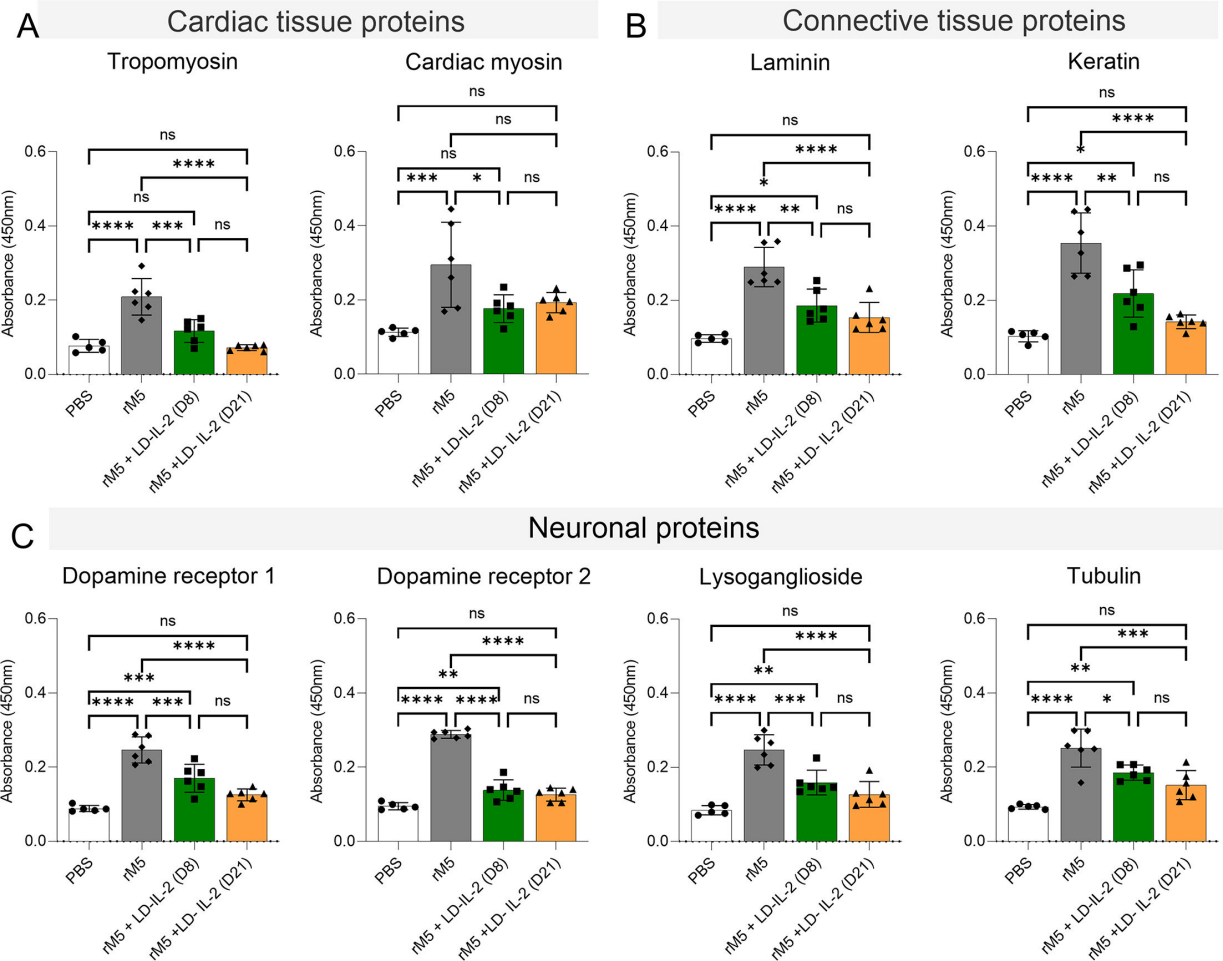
LD-IL-2 therapy reduces serum IgG cross-reactivity to host cardiac, connective tissue, and neuronal proteins. Serum IgG antibody levels were measured against (**A**) cardiac proteins (tropomyosin and cardiac myosin), (**B**) connective tissue proteins (laminin and keratin), and (**C**) neuronal proteins (dopamine receptors 1 and 2, lysoganglioside and tubulin). Absorbance values of Day-35 rat sera at 1:400 dilution are shown for all groups (*n* = 5–6 females/grp). Statistical analysis was performed by one-way ANOVA with Dunnett multiple comparison test. n.s. non-significant, *P  <  0.05, **P  <  0.01, ***P < 0.005, ****P <  0.001. Bars represent standard deviation (SD).

A key aspect of the RAV model is its ability to generate antibodies that cross-react with cardiac and neuronal tissue. To determine whether LD-IL-2 therapy can inhibit cross-reactive antibody production, we conducted enzyme-linked immunosorbent assay (ELISA) against cardiac myosin and tropomyosin (heart muscle), laminin (heart valve extracellular matrix), and dopamine receptors types 1 and 2, tubulin and lysoganglioside (brain). Although the sera from rM5-injected rats exhibited significant cross-reactivity with all tissue proteins compared to the negative control PBS group, sera from rM5-injected rats treated with LD-IL-2 showed no reactivity with tropomyosin and cardiac myosin (Fig. 2A). Similarly, LD-IL-2 also resulted in significant reduction in the levels of IgG antibodies recognizing connective and neuronal proteins (Fig. 2B and C). No significant difference was observed between both treatment groups, and LD-IL-2 treatment started at Day 21 resulted in antibody levels similar to the negative control group.

### LD-IL-2 therapy selectively expands Tregs in the lymph nodes draining the heart

**Fig 3:**
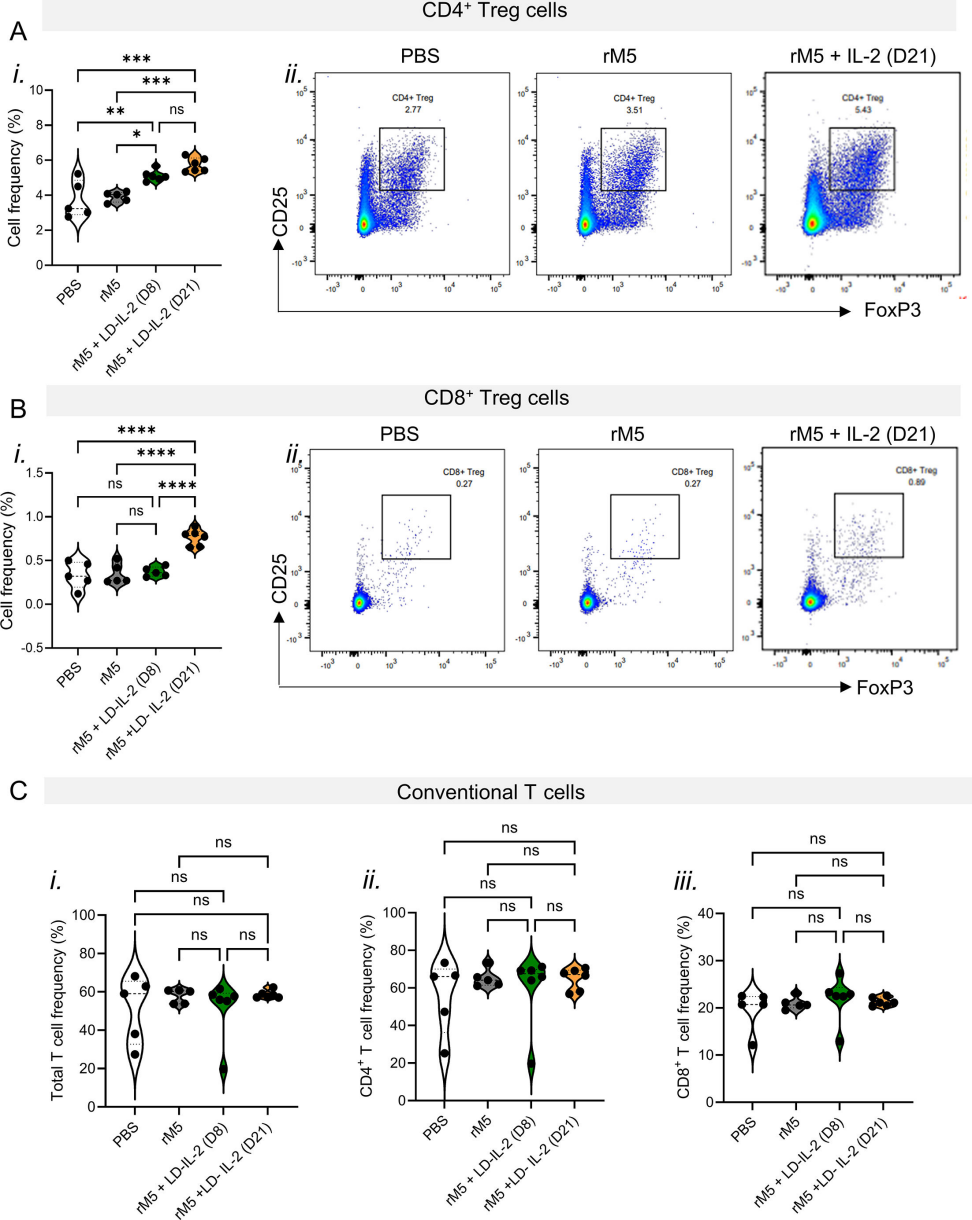
LD-IL-2 therapeutic efficacy is associated with a targeted increase in Tregs. (**A**) (*i*) Violin plot is used to show the proportion on CD4^+^ Treg (CD3^+^CD4^+^CD25^+^FoxP3^+^) in rats treated with PBS, rM5 only, and rM5 + LD-IL-2 (*n* = 5–6). Each symbol represents an individual rat analyzed. (*ii*) Representative gating strategy for defining CD4^+^ Treg by flow cytometry analysis. (**B**) (*i*) Proportion on CD8^+^ Treg (CD3^+^CD8^+^CD25^+^FoxP3^+^) in the different treatment groups. Each symbol represents an individual rat analyzed (*n* = 5–6 females/grp). (*ii*) Representative gating strategy for defining CD8^+^ Treg. (**C**) Violin plot is used to show the proportion of (*i*) total T cells within the lymphocyte population, along with (*ii*) CD4^+^ T cells within total T cells and (*iii*) CD8^+^ T cells within total T cells in rats treated with PBS, rM5 only, and rM5 + LD-IL-2. Each symbol represents an individual rat donor analyzed (*n* = 5–6 females/grp). Non‐parametric one‐way ANOVA test with Bonferroni correction. ns = non-significant, *P < 0.05, **P < 0.01, ***P < 0.005, ****P <  0.001.

Reduced Treg numbers or function have been implicated in the pathogenesis of various autoimmune diseases (29, 30), leading to the emergence of LD-IL-2 as a therapeutic strategy for selectively inducing Tregs while minimizing effects on immune effector cells (31). We used flow cytometry to investigate in the RAV model the impact of LD-IL-2 therapy on Tregs and conventional T cells from mediastinal lymph nodes. Strikingly, LD-IL-2 therapy resulted in 50% increase of classical Tregs (CD4^+^CD25^+^FoxP3^+^) in rats injected with rM5 (Fig. 3A). This is consistent with the expansion achieved in rodent models assessing efficacy of LD-IL-2 therapy for treating other autoimmune diseases (19). Additionally, an increase of rare CD8^+^ Tregs (CD8^+^CD25^+^FoxP3^+^) was observed when rats were treated with LD-IL-2 from Day 21, but not when they were treated from Day 8 (Fig. 3B).

To determine if the LD-IL-2 therapeutic regimen used in this study could also expand conventional T cells, we analyzed the frequencies of total T cells and the CD4^+^ and CD8^+^ T cell subsets. Notably, LD-IL-2 therapy did not alter the frequencies of conventional T cells but specifically expanded Tregs in the mediastinal lymph nodes (Fig. 3C), indicating that LD-IL-2 offers a targeted approach for managing post-streptococcal autoimmune complications.

## DISCUSSION

In our previous work utilizing the RAV model, we demonstrated that a single rM5 booster injection, combined with adjuvants at Day 7, induced mild myocarditis and valvulitis (32). Rats receiving further booster injections exhibited higher inflammatory scores, and a significant prolongation of the *P*–*R* interval on ECGs was only observed after the third booster with rM5, between Days 21 and 28 (32). Therefore, we propose that the LD-IL-2 therapeutic regimens employed in this study reversed the cardiac inflammation induced by rM5 exposure and prevented the onset of heart conduction abnormalities. Commencing LD-IL-2 therapy either early or later in the time course of rM5 protein exposure and hence disease onset was effective in treating any existing disease. It is possible that the timing of LD-IL-2 treatment in ARF may influence the effectiveness of LD-IL-2 therapy, depending on the stage and chronicity of the autoimmune process.

In RHD, chronic valvular disease begins with tissue cross-reactive antibodies that activate the valvular endothelium and trigger carditis. In Sydenham’s chorea, cross-reactive antibodies that transverse the compromised blood–brain barrier and bind to neuronal proteins in the basal ganglia are considered to be the cause for the onset of neurobehavioral symptoms (5, 33). Particularly, autoantibodies against dopamine receptors may lead to a receptor imbalance and induce greater sensitivity to dopamine signaling potentially leading to neuropsychiatric symptoms (34). Both IgG and IgM autoantibodies contribute to the pathogenesis of ARF and serve as biomarkers for the autoimmune sequelae of Strep A infections (35–37). While our focus for the ELISAs was specifically on IgG detection, the antibody used in this study is also known to recognize the light chains of other rat immunoglobulins. Therefore, we cannot exclude the potential role of IgM antibodies targeting autoantigens in this model. LD-IL-2 can indirectly affect B cell differentiation and antibody production. By promoting a regulatory environment through Tregs, LD-IL-2 helps to control the differentiation of B cells into antibody-secreting plasma cells that are enriched for a regulatory B cell signature (38), thereby reducing the production of self-reactive antibodies. Besides, LD-IL-2 suppresses the activation and differentiation of T follicular helper cells (39, 40). This helps to limit excessive germinal center responses that contribute to autoantibody production and promotes immune tolerance.

While key modifiable risk factors for ARF include household crowding, poor access to primary healthcare, and untreated Strep A infections (41, 42), extensive investigations have been conducted to identify host susceptibility factors, including human leukocyte antigens, B cell alloantigens, and cytokine genes. Some studies have found notable associations between genetic factors and ARF, but the results are often inconsistent and contradictory (43). In general, human leukocyte antigen (HLA) class II molecules appear to have a closer association with increased risk of ARF and RHD than class I molecules; however, no single HLA haplotype or combination exists that is consistently associated with susceptibility. One theory suggests that the structural similarity between streptococcal antigens and HLA molecules leads to mimicry, triggering an abnormal immune response (44). Although the genetic component of human ARF/RHD remains unclear, the Lewis rat strain is genetically predisposed to autoimmune conditions due to specific host factors that contribute to immune dysregulation. Notably, most autoimmune-associated CD4^+^ T cell responses in Lewis rats are MHC class II RT1.BL-restricted (45). As a result, our study investigating the use of LD-IL-2 therapy to reverse the autoimmune process is particularly compelling when applied to a genetically susceptible rat model. Lewis rats have commonly been used to develop models for studying autoimmune diseases, including RHD (46, 47), Sydenham’s chorea (46, 48), multiple sclerosis (49), autoimmune encephalitis (50), and uveitis (51).

Here, we demonstrate an increase in both classical Tregs and CD8^+^ Tregs following treatment with LD-IL-2. In contrast to classical Tregs, the origin and roles of CD8^+^ Tregs in the pathogenesis of autoimmune diseases are less well understood. While naturally occurring CD8^+^ Tregs are rare, they can be induced *in vivo* (52) and are generally considered to maintain less stable expression of FoxP3 than classical Tregs (53, 54). Nonetheless, CD8^+^ Tregs show heightened sensitivity to IL-2 for proliferation compared to CD8^+^ effector T cells and have been induced by LD-IL-2 therapy in both mice and humans (22, 55, 56). Similar to their CD4^+^ counterpart, CD8^+^ Tregs have been shown to play important roles in immune regulation by suppressing autoimmune and inflammatory responses through a variety of mechanisms, including cytokine secretion, cell-to-cell contact, induction of a tolerogenic phenotype in antigen-presenting cells that can subsequently promote classical Tregs, and cytotoxic activity (57, 58). Despite Tregs being impaired during RHD (10, 11), stimulation with streptococcal and staphylococcal superantigens can induce human CD8^+^ Tregs and classical Tregs (59–61). This suggests that Treg induction during acute bacterial infections may facilitate immune evasion and contribute to disease pathogenesis. However, data on the effects of superantigens on rat T cells remain limited (62). Recently, a study involving participants in a Strep A human challenge trial showed a decreased number of circulating Tregs during acute pharyngitis (63). While it is plausible that Tregs migrated to the site of infection to modulate the inflammatory responses, further investigation is needed. Ultimately, it is possible that exposure to Strep A superantigens could boost Treg responses following LD-IL-2 therapy, potentially suppressing the cardiac inflammatory events that lead to RHD.

Tregs constitutively express high levels of the heterotrimeric high-affinity IL-2 receptor (IL-2R) complex, with the highest expression of its α chain (CD25), making them particularly sensitive to even small amounts of IL-2 in the body (64). A genetic deficiency in the IL-2/IL-2R pathway may lead to systemic autoimmunity (65). Due to this unique property, LD-IL-2 therapy has become an emerging immunotherapeutic approach for treating certain autoimmune conditions. In this study, we did not observe increase of CD8^+^ Tregs at Day 35 when rats were treated with LD-IL-2 therapy starting from Day 8. This lack of induction is likely due to the instability of FoxP3 expression in induced Tregs (66), which could not be sustained until the experiment endpoint. Although LD-IL-2 expanded Tregs and reduced disease severity in several autoimmune diseases (13, 14, 67), IL-2 is also a growth factor for potentially pathogenic effector T cells due to the widespread expression of IL-2R subunits. Thus, IL-2’s dual role in promoting both tolerance and activation makes predicting its therapeutic effects challenging. Moreover, LD-IL-2 has been demonstrated to exhibit limited or no therapeutic efficacy in alloimmune conditions (68) as well as heterogeneity in clinical responsiveness in various autoimmune diseases (21).

Here, we experimentally demonstrated that subcutaneous administration of LD-IL-2 effectively mitigated post-streptococcal autoimmune complications. This treatment improved functional cardiac activity, reduced cardiac inflammation, and decreased heart and brain cross-reactive antibody responses in the RAV model. The short half-life of IL-2 requires repetitive injections to sustain therapeutic levels of Tregs (69). While secondary prophylaxis for RHD with penicillin requires monthly hospital visits for intramuscular injections over a period of up to 10 years, patients receiving subcutaneous LD-IL-2 treatment could instead be provided with a long-term supply of reconstituted IL-2 in single-use syringes for home storage and administration (70). This approach would eliminate the need for frequent hospital visits, improving accessibility, particularly for individuals in rural and remote communities with limited access to healthcare facilities.

Our findings indicate that LD-IL-2 therapy may effectively treat ARF, potentially improving clinical outcomes and reducing the cardiac complications associated with RHD. Further studies are required to determine the duration of the LD-IL-2 therapeutic effect and whether this regimen has the potential to prevent abnormal responses following recurrent exposure to streptococcal antigens. Nevertheless, this therapy has the potential to replace the need for standard monthly penicillin injections.

## MATERIALS AND METHODS

### Animals

All experimental protocols involving animals were approved by the Animal Ethics Committee of the University of New England (UNE) (ARA23-009). Female Lewis rats (LEW/SsN; Albino:a,h,c:RT^1^) aged 4–6 weeks were purchased from the Centre for Animal Research and Teaching at UNE and acclimatized for 5 days prior to experiments.

### Preparation of recombinant M protein from Strep A, type 5 (rM5)

Recombinant M5 protein of Strep A (rM5) was cloned and purified as described previously (71). Briefly, Strep A *emm5* was cloned into pREP4 vector and expressed in *Escherichia coli* BL21. rM5 protein preparations were purified using Ni-NTA resin. Lipopolysaccharide (LPS) contamination in recombinant protein preparations was removed by Triton X-114-assisted LPS extraction as previously described (72). The rM5 protein preparations were determined to be free of LPS using Pierce Chromogenic Endotoxin Quantification Kit (ThermoFisher, USA) as per manufacturer’s instructions.

### Induction of autoimmune carditis and valvulitis

For induction of carditis, rats were injected with 0.5 mg/100 µL of Strep A rM5 protein emulsified in complete Freund’s adjuvant (CFA) or PBS emulsified in CFA. Baseline ECG was performed prior to injection. All priming injections were performed under isoflurane inhalation anesthesia (5% induction and 2% maintenance) in the hock as described previously (73). On Days 1 and 3, all rats were intraperitonially injected with 300 ng of *Bordetella pertussis* toxin (Gibco, USA) in 200 µL PBS. Rats were boosted with respective antigens in IFA on the flank on Days 7, 14, and 21. The rats were euthanized on Day 35 from the day of primary injection with the overdose of sodium pentobarbital (260 mg/kg, i.p), blood was collected by cardiac puncture, and heart tissue was collected and fixed with 4% paraformaldehyde for histopathological analysis. Heart draining mediastinal lymph nodes were collected for flow cytometry analysis.

### LD-IL-2 treatment

Age-matched female Lewis rats were divided into four treatment groups (*n* = 5/6): (i) PBS, (ii) Strep A rM5, (iii) Strep A rM5 + IL-2 (Day 8), and (iv) Strep A rM5 + IL-2 (Day 21). Recombinant human IL-2 (#200-02, Peprotech, USA) was reconstituted in 100 mM acetic acid and further diluted in PBS containing 0.1% bovine serum albumin to achieve 20,000 IU concentration. Rats in group iii received subcutanous injections of LD-IL-2 from Day 8 of primary injection to Day 24 on every other day. Rats in group iv received subcutanous injections of LD-IL-2 from Day 21 of primary injection to Day 34 on every other day (Fig. 1A).

### Electrocardiography

ECG traces were recorded for 1–2 min using Bio Amp with PowerLab data acquisition system (ADInstruments, USA) in all rats prior to injection and a day before euthanasia to assess the conduction abnormalities in the heart. Peak values of *P* and *R* point at three different segments of ECG from each rat that was individually extracted and analyzed (46).

### Histopathological analysis of cardiac tissue

To examine the extent of inflammation, formalin-fixed paraffin-embedded cardiac tissue was processed, embedded in paraffin, sectioned, and stained with Harris H&E using standard procedures as previously described (71). Slides were examined microscopically for infiltration of mononuclear cells as evidence of myocarditis or valvulitis. The extent of inflammation was expressed as a “carditis score” based on the number of mononuclear inflammatory cells and focal lesions from each rat (26). The scoring system assesses and scores valvular and myocardial sections (five randomly selected areas per animal) from the different groups using a semi-quantitative scoring system.

### Detection of tissue-reactive serum antibodies

Serum IgG antibodies against purified host proteins including cardiac myosin, tropomyosin, laminin, keratin, dopamine receptors 1 and 2, lysoganglioside GM1 and tubulin (Sigma, USA) were determined using indirect ELISA as described previously (25). Briefly purified proteins were coated onto Maxisorp 96-well plates (Nunc, USA) in carbonate–bicarbonate buffer. Plates were blocked and incubated with individual rat sera in duplicate at 1:400 dilution. Following repeated washing of the plates, each well was incubated with 100 µL of goat anti-rat IgG HRP conjugated secondary antibody (1:5,000; Jackson Immunoresearch, USA) for an hour at room temperature. Each well was washed and incubated for 20 min with 100 µL of SIGMAFAST OPD (Sigma, USA). Absorbance was measured at 450 nm using SpectraMax M2/M2e (Molecular Devices, USA).

### Multiparametric flow cytometry for analysis of circulating Tregs

Mediastinal lymph nodes were dissociated into single-cell suspension using a plunger from a sterile syringe. Red blood cells were lysed with ACK lysis buffer and washed with RPMI (Thermo Fisher Scientific, AUS). The cell populations were determined by flow cytometry. The cells were surface-stained with a master mix containing dead cell exclusion dye (Zombie Violet), CD4-APC-Cy7, CD3-PerCP, CD8-PE-Cy7, and CD25-APC. Cells were stained on ice in the dark for 40 min. Intranuclear staining for FoxP3-PE was achieved using FoxP3 Fix/Perm Buffer Set for nuclear staining (BioLegend, San Diego, California, USA). Samples were washed in PBS and acquired by a BD Fortessa multiparametric flow cytometer (BD Biosciences, Franklin Lakes, New Jersey, USA). Data were analyzed using FlowJo V.10.7 (BD).

### Statistical analysis

Descriptive statistics of results were determined using GraphPad Prism version 8.0. Antibody titer data are presented as geometric mean. One-way analysis of variance (ANOVA) with Dunnett post hoc method for multiple comparisons was used for pairwise comparisons. Results were defined as significant with *P* < 0.05 or <0.01. Two-way ANOVA was used to assess ECG differences between rats. All data with *P* < 0.05 were considered significant.
